# Should We Use an Orphan Graft?

**Published:** 2017-11-01

**Authors:** A. Baskıran, F. Ozdemir, B. Barut, V. Ince, C. Koc, V. Ersan, S. Karakas, K. Kutluturk, S. Yılmaz

**Affiliations:** 1 Department of Surgery, Inonu University School of Medicine, Turgut Ozal Medical Center, Malatya, Turkey; 2 Department of Radiology, Inonu University School of Medicine, Turgut Ozal Medical Center, Malatya, Turkey

The intra-operative death of a liver transplant recipient is a devastating complication that occurs very infrequently. If the liver graft is exactly procured from the living donor at the same time, it is impossible to transplant this graft to the intended recipient; this liver graft is called an “orphan graft” [3] The appropriate allocation of the orphan graft requires special consideration and raises several important questions. Herein, we report on the outcomes of two orphan grafts that were no longer transplanted to the intended recipients. There are no set rules for how to manage these situations. 

Our first potential recipient was a 65-year-old woman who had end-stage liver disease due to cryptogenic cirrhosis. Her Child-Turcotte-Pugh and MELD scores were C13 and 27, respectively. The other intended recipient was a 41-year-old man, also with end-stage liver disease due to hepatitis B virus-related cirrhosis. His Child-Turcotte-Pugh and MELD scores were C12 and 21, respectively (Table 1). Both patients were evaluated for living-donor liver transplantation. The standard pretransplantation cardiopulmonary evaluations were completed in the two recipients. Electrocardiogram, stress electrocardiogram, myocardial scintigraphy, pulmonary function tests, and also cardiology consultations were requested for both patients where no pathological findings were detected, and both recipients were found to be suitable candidates for liver transplantation.

**Table 1:** Characteristics of the two recipients

Parameter	Recipient 1	Recipient 2
Age (yrs)	65	41
Sex	Female	Male
Blood group	A Rh^+^	B Rh^+^
MELD Score	27	21
Child Score	C13	C12
Ejection fraction	55%	60%
Pulmonary artery pressure (mm Hg)	30	30
Disease	Criptogenic cirhosis	HBV-related liver disease
Cause of death	Myocardial infarction	Myocardial infarction

Living donors underwent our standardized multistep evaluation protocol for the right lobe liver donation. The donors, 22- and 24-year-old healthy individuals, were properly informed of the risks of both surgical procedures (donor and recipient) and also of potential complications.

The donors and recipients operations were performed according to the techniques already described by our group. The donors hepatectomy and the recipients total hepatectomy were completed almost simultaneously by two surgical teams.

Almost 1600 liver transplantations were performed at Liver Transplantation Institute of Inonu University between 2002 and 2015; 329 (20.5%) of them were cadaveric liver transplantation and the remaining 1270 (79.5%) were living-donor liver transplantations. There were only two orphan grafts that were used according to the legal procedures.

Two recipients died during the anhepatic phase, of myocardial infarction even though we did not observe any significant cardiopulmonary diseases during preoperative evaluation period. In fact, both of the recipients were found to be suitable candidates for liver transplantation. Review of the preoperative evaluation and of the intraoperative anaesthesia records for each recipient showed nothing abnormal until the time of cardiac arrest. Unfortunately, after all the efforts made to keep them alive we could not make it. At the same time, donor hepatectomy operation was completed and we had two suitable grafts.

Should we use these orphan grafts for other suitable patients? First of all, we talked to the recipients and donors families. They accepted to utilize the orphan grafts for other patients waiting in the transplant list. After taking the written informed consents of the families, we informed the national organ network sharing system about the situation who told us to use the grafts for patients waiting in our list because there were no urgent cases on the national waiting list at the same time. We then used the orphan grafts for two suitable recipients from our waiting list.

Fernando, *et al*, reported the first orphan graft in Mexico in 1999 [1]. He encountered this situation as an ethical and medical problem. He emphasized the need for new laws about orphan grafts. We believe that legal regulations for utilizing orphan grafts should be prepared. Legal basis can disburden both the transplant professionals and the family.

Siegler, *et al*, reported a study about orphan grafts. They asked 22 transplant surgeons and four hepatologists that “what should we do when orphan graft occurs?” Twenty-six of the interviewed surgeons (100%) said that the surgical team should reallocate the graft [2]. However, there are different opinions about the distrubition of the orphan graft and family approval. Nowadays, considering the gap between the supply and the demand for liver transplantation, every organ is valuable, so we believe to use every suitable organ including an orphan graft.

Nadalin, *et al*, reported four healthy donors having the rare condition of *hepar divisum* after intraoperative death of the intended recipients during right lobe adult liver transplantation [4]. Death of a recipient after the transection of the donor biliary tract is a complicated problem for the donors if the individual has more than one biliary duct. The authors reported that instead of reconstructing the biliary tracts of donors, it would be better to create orphan grafts for the donors having continious biliary tract complications [4].

Wachs, *et al*, reported that a simple solution for the problem of orphan grafts is to avoid this situation [3]. It is critical to make an effort to prevent the occurence of an orphan graft, but it should be kept in mind to have a guide in case of having an orphan graft. It was not difficult to talk about the orphan graft with the donors and the recipients families at our clinic. In order to rescue another patient, we told them the possibility of saving other lives.

We have gained some important information after orphan grafts. Preoperative donor approval against the possibilty of orphan graft formation should be undertaken. Detailed preoperative evaluation for the recipient should be performed. Donor and recipient surgery should be performed simultaneously. Surgery and anesthesia team should be experienced. Finally, if an orphan graft occurs, we should take approval from the donors and recipients families for allocation.
